# Jasmonic acid and methyl jasmonate modulate growth, photosynthetic activity and expression of photosystem II subunit genes in *Brassica oleracea* L

**DOI:** 10.1038/s41598-020-65309-1

**Published:** 2020-06-09

**Authors:** Geetika Sirhindi, Ruqia Mushtaq, Sarvajeet Singh Gill, Poonam Sharma, Elsayed F. Abd_Allah, Parvaiz Ahmad

**Affiliations:** 1 Plant Physiology Laboratory, Department of Botany, Punjabi University, Patiala, 147002 Punjab India; 2 Stress Physiology and Molecular Biology Lab, Centre for Biotechnology, Maharshi Dayanand University, Rohtak, 124 001 Haryana India; 3 Department of Plant Production, Faculty of Food & Agricultural Sciences, King Saud University, Riyadh, Saudi Arabia; 4 Botany and Microbiology Department, College of Science, King Saud University, Riyadh, Saudi Arabia; 5 Department of Botany, S.P. College Srinagar, Jammu and Kashmir, India

**Keywords:** Jasmonic acid, Plant physiology

## Abstract

The effects of jasmonic acid (JA) and methyl jasmonate (Me-JA) on photosynthetic efficiency and expression of some photosystem (PSII) related in different cultivars of *Brassica oleracea* L. (var. *italica, capitata*, and *botrytis*) were investigated. Plants raised from seeds subjected to a pre-sowing soaking treatment of varying concentrations of JA and Me-JA showed enhanced photosynthetic efficiency in terms of qP and chlorophyll fluorescence. Maximum quantum efficiency of PSII (*F*v/*F*m) was increased over that in the control seedlings. This enhancement was more pronounced in the Me-JA-treated seedlings compared to that in JA-treated ones. The expression of PSII genes was differentially regulated among the three varieties of *B. oleracea*. The gene *PsbI* up-upregulated in var. *botrytis* after treatment of JA and Me-JA, whereas *PsbL* up-regulated in *capitata* and *botrytis* after supplementation of JA. The gene *PsbM* showed many fold enhancements in these expressions in *italica* and *botrytis* after treatment with JA. However, the expression of the gene *PsbM* increased by both JA and Me-JA treatments. *PsbTc(p)* and *PsbTc(n)* were also found to be differentially expressed which revealed specificity with the variety chosen as well as JA or Me-JA treatments. The RuBP carboxylase activity remained unaffected by either JA or Me-JA supplementation in all three varieties of *B. oleracea* L. The data suggest that exogenous application of JA and Me-JA to seeds before germination could influence the assembly, stability, and repair of PS II in the three varieties of *B. oleracea* examined. Furthermore, this improvement in the PS II machinery enhanced the photosynthetic efficiency of the system and improved the photosynthetic productivity in terms of saccharides accumulation.

## Introduction

Plant metabolism is a consequence of dynamic interactions among all its components, which are involved in an intricate array of events. Hormones affect the intricate interactions of PSII proteins with pigments associated with light harvesting complexes in order to generate free energy. The gene *PsbI* is primarily associated with the structural stabilization and proper functioning of the PS II reaction centre. PS II is involved in electron transport during photolysis of water to the plastoquinone, the protein involved in proton pumping. The gene *PsbI*, associated with the PSII reaction centre, encodes a 4.8 kDa polypeptide, and *PsbI* is involved in stabilizing the dimeric as well as the super-complexes of PSII for proper functioning^1^. In *Synechocystis* sp. PCC 6803, specifically during the early stages of *de novo* PSII assembly, PsbI protein binds to nascent D1 protein, which further validates the role of PsbI in stabilizing the association of CP43 to the PSII core complexes^2^. The PsbL together with PsbM and PsbTc are located in the reaction centre of the D2 protein at the monomer-monomer interface of PSII complex proximal to the Q_A_ site^3–6^. In order to restore the activity of Q_A_ on the acceptor side, the participation of PsbI is essential^7,8^. PsbL prevents the reduction of PSII by the back flow of electron from plastoquinol, because it acts on the acceptor side^4–6,9^. Studies on *Synechocystis* 6803^10^ and tobacco^11^ demonstrated the loss of photoautotrophic growth as well as PSII activity by inactivation of PsbL. The PsbM protein in the PSII reaction centre has been detected in both lower organisms such as cyanobacteria, *Chlamydomonas*, and in higher plants such as *Arabidopsis*. PsbM in tobacco is implicated in the flow of electrons within and outwards of PSII^12^. A leucine zipper connecting the two PsbM proteins is responsible for holding the two PSII subunits together, thus PsbM plays a major role in dimerization. The intrinsic polypeptide, PsbTc, encoded by the chloroplast DNA has been conserved from cyanobacteria through higher plants. When the wild type and a *PsbTc* mutant in *Synechocystis* sp. 6803 were compared, the mutant suffered a decreased oxygen release and a reduced PSII assembly apart from very slow doubling time^13^. Inactivation of the *PsbTc* gene accelerates photodamage, as it acts on the acceptor side of PSII reaction centre^14^. The PsbTc(n) is a soluble protein encoded by nuclear genes, and not much is known about its function. Similarly, PsbTc(p) peptide, encoded by the plastid DNA and present in the PSII reaction centre has not been explored.

Plant growth and development are coordinated by both external and internal signals. To date several regulators of plant growth and development have been identified. Jasmonates are one of the potential plant growth regulators, which comprise small molecules, including free JA, and its conjugates Me-JA and jasmonyl isoleucine^15^. MeJA affects photosynthesis-related activities and antioxidants in plants by modulating protein profile^16^. Jasmonates are derived from chloroplast-based lipid, linolenic acid. They are synthesised via the octadecanoid pathway responsible for the formation of 12-oxo phytodienoic acid (OPDA), a JA precursor, which is further modified enzymatically to form various derivatives, including MeJA^17–19^. Jasmonates lead to the accumulation of chlorophyll, carotenoids, and other pigments as well as the increased PSII efficiency in *Saxifraga longifolia*^20^. Drastic reduction in the expression of ribulose-1,5-bisphosphate carboxylase/oxygenase (RuBisCO) subunits in *Oryza sativa* due to jasmonates has also been reported^21^.

*Brassica oleracea* L. var. *capitata, botrytis*, and *italica* comprise important vegetable crops such as broccoli, cauliflower, kale, and cabbage^22^. A diet rich in cruciferous vegetables lowers the risk of several human cancers^23,24^. Due to its considerable consumption and wide distribution as a vegetable, it has been chosen as a model crop in this research. MeJA is involved in the alleviation of adverse effects of drought stress, thus imparting resistance to *B. oleracea* by promoting defense-related metabolism^25^. However, not much is known about the JA-regulated moniker changes during photosynthesis in *B. oleracea* L. On the other hand, exogenous application of JA and MeJA modulates photosynthesis, pigmentation, photosynthetic efficiency, and expression of PSII genes. In this study, we examined changes in photosynthesis, photochemistry of PSII RC, and genes associated directly with the stabilization, proper functioning and maintenance of PS II by the exogenous application of JA and MeJA in *B. oleracea* L. var. *capitata, botrytis*, and *italica*. In addition, sugar forms and its levels were also monitored as they affect plant growth and development.

## Results

### Pre-treatment of JA and MeJA improves growth

*Brassica oleracea* var. *capitata, botrytis*, and *italica* seedlings treated with different levels of JA and Me-JA, responded variably in terms of root growth, shoot growth, fresh weight, dry weight, moisture content and percent biomass (Table 1). An increase of 52.69% and 43.11% in root length of *B. oleracea* var. *capitata* seedlings was observed when treated with 1 µM JA and MeJA, respectively. An increase by 58.68% and 50.29% was observed with 1 nM JA and Me-JA, respectively, as compared to the control seedlings of all cultivars. The root length increased further by 9.49% and 20.88% in response to 1 nM JA and MeJA, respectively, in comparison to that of the control seedlings. On the contrary, there was no change in root length in *B. oleracea* var. *italica* with JA supply, but root length increased to 34.61% in exposure to 1 pM MeJA as compared to that in the control seedlings (Table 1).

**Table 1:** Effects of Jasmonic acid (JA) and Methyl jasmonate (Me-JA) on the root length, shoot length, fresh weight, dry weight, moisture content and percentage biomass of *Brassica oleracea* L. var*. italica, capitata* and *botrytis*. Values are means ± SD of three independent replications (n = 3). Different letters (a–e) within the column indicate statistically significant differences among the treatments, according to Tukey’s test at (P < 0.05).

Parameters	Varieties	Control	1 µM JA	1 nM JA	1 pM JA	1 µM MeJA	1 nMMeJA	1 pMMeJA
Root Length (cm)	Italica	6.07 ± 0.40^b^	5.17 ± 0.15^c^	3.27 ± 0.25^d^	4.33 ± 0.32 ^cd^	4.50 ± 0.44 ^cd^	3.17 ± 0.29^de^	8.17 ± 0.29^a^
	Capitata	5.57 ± 0.60^bc^	8.50 ± 0.52^a^	8.83 ± 0.21^a^	6.53 ± 0.42^b^	7.97 ± 0.40^a^	8.37 ± 0.32^a^	8.07 ± 0.12^a^
	Botrytis	5.27 ± 0.71^a^	4.27 ± 0.21^b^	5.77 ± 0.15^a^	5.40 ± 0.20^a^	5.53 ± 0.12^a^	6.37 ± 0.25^a^	5.63 ± 0.25^a^
Shoot Length (cm)	Italica	6.13 ± 0.12^a^	4.17 ± 0.76^ab^	5.70 ± 0.26^a^	5.87 ± 0.31^a^	5.00 ± 0.10^a^	6.03 ± 0.15^a^	6.43 ± 0.67^a^
	Capitata	5.13 ± 0.55^a^	5.00 ± 0.20^a^	4.90 ± 0.26^a^	4.63 ± 0.42^a^	5.00 ± 0.66^a^	4.80 ± 0.20^a^	4.70 ± 0.26^a^
	Botrytis	5.20 ± 0.10^a^	3.97 ± 0.31^ab^	3.67 ± 0.15^ab^	4.17 ± 0.31^ab^	4.17 ± 0.38^ab^	4.60 ± 0.10^a^	3.73 ± 0.21^ab^
Fresh Weight (g)	Italica	0.645 ± 0.004^g^	0.661 ± 0.004^f^	0.732 ± 0.003^c^	0.768 ± 0.003^b^	0.688 ± 0.003^e^	0.721 ± 0.004^d^	0.812 ± 0.004^a^
	Capitata	0.967 ± 0.006^efg^	1.073 ± 0.004^a^	0.986 ± 0.005^e^	1.026 ± 0.006^c^	1.060 ± 0.003^b^	1.005 ± 0.004^d^	0.979 ± 0.003^ef^
	Botrytis	0.480 ± 0.002^f^	0.607 ± 0.001^a^	0.491 ± 0.004^e^	0.587 ± 0.002^bc^	0.529 ± 0.002^d^	0.589 ± 0.002^b^	0.548 ± 0.004^c^
Dry Weight (g)	Italica	0.029 ± 0.002^cde^	0.037 ± 0.002^bc^	0.033 ± 0.002^bcd^	0.038 ± 0.001^bc^	0.035 ± 0.001^bc^	0.038 ± 0.002^b^	0.048 ± 0.001^a^
	Capitata	0.045 ± 0.002^a^	0.051 ± 0.002^a^	0.044 ± 0.002^a^	0.046 ± 0.002^a^	0.052 ± 0.003^a^	0.048 ± 0.002^a^	0.046 ± 0.002^a^
	Botrytis	0.025 ± 0.002^a^	0.023 ± 0.001^a^	0.022 ± 0.002^a^	0.024 ± 0.001^a^	0.022 ± 0.001^a^	0.027 ± 0.003^a^	0.025 ± 0.001^a^
Moisture content (%)	Italica	95.45 ± 0.29^a^	94.45 ± 0.22^a^	95.53 ± 0.23^a^	95.05 ± 0.11^a^	94.92 ± 0.12^a^	94.69 ± 0.18^a^	94.09 ± 0.13^a^
	Capitata	95.35 ± 0.18^a^	95.28 ± 0.13^a^	95.50 ± 0.19^a^	95.52 ± 0.17^a^	95.13 ± 0.25^a^	95.23 ± 0.18^a^	95.30 ± 0.19^a^
	Botrytis	94.79 ± 0.43^a^	96.21 ± 0.16^a^	95.52 ± 0.39^a^	95.85 ± 0.25^a^	95.84 ± 0.18^a^	95.30 ± 0.52^a^	95.43 ± 0.20^a^
Percentage biomass (%)	Italica	4.55 ± 0.29^ab^	5.55 ± 0.22^a^	4.47 ± 0.23^ab^	4.95 ± 0.11^ab^	5.08 ± 0.12^ab^	5.31 ± 0.18^ab^	5.91 ± 0.13^a^
	Capitata	4.65 ± 0.18^a^	4.72 ± 0.13^a^	4.50 ± 0.19^a^	4.48 ± 0.17^a^	4.87 ± 0.25^a^	4.77 ± 0.18^a^	4.70 ± 0.19^a^
	Botrytis	5.21 ± 0.43^a^	3.79 ± 0.16^a^	4.48 ± 0.39^a^	4.15 ± 0.25^a^	4.16 ± 0.18^a^	4.70 ± 0.52^a^	4.57 ± 0.20^a^

More specifically, in *B. oleracea* var. *italica*, the shoot length decreased by 32.07% and 18.48%, respectively, when treated with 1 µM JA or MeJA, compared to the control. When JA and MeJA were diluted to 1 nM, the inhibitory effects were minimal, and with a further dilution to 1 pM, 4.89% increase in shoot length was observed when compared to the control. On the other hand, in *B. oleracea* var. *capitata* a consistent decline in shoot length was observed for both JA and MeJA treatments. Moreover, a proportionate reduction in shoot length in the order of 2.60%, 4.55%, and 9.74%, respectively, due to treatment with 1 µM, 1 nM and 1 pM Me-JA and by 2.60%, 6.49% and 8.44%, respectively, with 1 µM, 1 nM and 1 pM JA, when compared to the control seedlings (Table 1). The seedling biomass in terms of fresh weight and dry weight (Table 1) fluctuated depending on the concentrations of JA or MeJA in *B. oleracea* var. *capitata*. The treatment with both JA and MeJA at 1 µM improved the fresh weight by 9.58% and 10.89%, respectively, when compared with that of the control, while dry weight increased by 12.59% and 14.81% due to the treatment with 1 µM JA and MeJA, respectively, when compared to the control. Pre-treatment of JA and MeJA increased fresh weight of *B. oleracea* var. *italica*, but dry weight increased only with MeJA treatments at concentrations of 1 µM, 1 nM and 1 pM, by 19.32%, 30.68% and 63.63%, respectively, when compared to the control. Addition of 1 pM JA to *B. oleracea* var. *italica* improved its dry weight by 29.54%. Our data showed no change in dry weight of *B. oleracea* var. *botrytis* seedlings at all concentrations of JA and MeJA. A slight increase of 10.66% in dry weight was observed when treated with 1 nM MeJA. The moisture content declined in *B. oleracea* var. *italica* and *capitata* after pre-treatment with JA and MeJA in a dose-dependent manner. The moisture levels declined by 1.05% and 0.08% due to treatment with 1 µM JA in *B. oleracea* var. *italica* and *capitata*, respectively, when compared to the control. However, when *B. oleracea* var. *botrytis* seedlings were treated with 1 µM JA and MeJA, a slight increase in moisture content by 1.49% and 1.08%, respectively, was observed when compared to untreated control. In general, moisture content declined in all three cultivars when treated with JA and MeJA (Table 1). Percent biomass (Table 1) of seedlings of all three cultivars increased. The significant decline by 20.16% and 27.26% in the seedlings raised from 1 µM treatment of MeJA and JA, respectively, was recorded in *Botrytis* seedlings compared with the control. *B. oleracea* var. *italica* seedlings treated with MeJA produced increased biomass by 11.77%, 16.81% and 29.95%, when treated with 1 µM to 1 nM and 1 pM, respectively. In *B. oleracea* var. *italica* seedlings, percent biomass was increased by 21.94% and 29.95%, when treated with 1 µM JA and 1 pM MeJA, respectively, when compared to the untreated control seedlings. *B. oleracea* var. *capitata* seedlings had no change in percent biomass with either of the treatments.

### Seed priming with JA and MeJA maintains chlorophyll pigments, chl a/b, and carotenoids

Total chlorophyll content in *B. oleracea* var. *italica* underwent a minimal change, with only a slight increase of 7.80% and 5.62% when treated with 1 pM JA and 1 nM JA, respectively, and when compared with that of the untreated control seedlings (Table 2). Reduced total chlorophyll content was observed in 1 µM JA (1.63%), and in 1 nM Me-JA (6.49%). In case of *B. oleracea* var. *capitata*, all the JA treatments (except 1 pM JA) and Me-JA increased the total chlorophyll content, with the highest increase of 5.73% observed with 1 nM JA treatment, when compared to the control seedlings (Table 2). Total chlorophyll content of *B. oleracea* var. *botrytis* increased with all JA treatments as presented in Table 2. The seedlings treated with 1 µM JA exhibited increased total chlorophyll content (1.09 ± 0.04 mg/g FW), although it was not significant in comparison to that in the control seedlings treated with distilled water (1.03 ± 0.004 mg/g FW total chlorophyll content). Treatment with Me-JA led to a general decrease in the total chlorophyll content, except 1 pM Me-JA treatment that resulted in a 9.24% increase when compared to the control seedlings. Table 2 shows that chl a/b ratio increased in seed priming with JA and MeJA treatments in *B. oleracea* var. *botrytis* and *italica*. In *B. oleracea* var. *italica* it increased by 5.26% and 23.22%, when primed with 1 pM JA and 1 pM Me-JA, respectively, as compared to that in the control seedlings. In *B. oleracea* var. *capitata* similar results with JA priming were experienced. Only 1 nM JA priming led to a decrease in chl a/b ratio (5.81%), but the treatment with 1 µM and 1 pM JA increased this ratio by 3.32% and 9.11%, when compared with the control. Me-JA at all three concentrations tested led to a decrease in chl a/b ratio. In *B. oleracea* var. *botrytis* both JA and Me-JA increased the chl a/b ratio except for 1 µM JA. In the case of JA priming, 1 nM and 1pM JA increased the chl a/b ratio to almost the same level, which was 5.36% and 5.51%, respectively, as compared to that in the control seedlings. A maximum increase of 13.5% in chl a/b ratio was noted in 1 µM Me-JA supplemented seedlings as compared to the control seedlings.

**Table 2:** Effects of Jasmonic acid (JA) and Methyl jasmonate (Me-JA) on the total chlorophyll, Chl a/b ratio, and total carotenoids of *Brassica oleracea* L. var*.italica, capitata* and *botrytis*. Values are means ± SD of three independent replications (n = 3). Different letters (a–e) within the column indicate statistically significant differences among the treatments, according to Tukey’s test at (P < 0.05).

Parameters	Varieties	Control	1 µM JA	1 nM JA	1 pM JA	1 µM MeJA	1 nM MeJA	1 pM MeJA
Total chlorophyll (µg g^−1^FW)	Italica	0.66 ± 0.007^ab^	0.65 ± 0.009^ab^	0.70 ± 0.016^a^	0.72 ± 0.013^a^	0.68 ± 0.020^ab^	0.62 ± 0.007^ab^	0.67 ± 0.010^ab^
	Capitata	1.09 ± 0.02^a^	1.10 ± 0.02^a^	1.15 ± 0.00^a^	1.09 ± 0.02^a^	1.10 ± 0.02^a^	1.13 ± 0.01^a^	1.12 ± 0.03^a^
	Botrytis	1.03 ± 0.004^a^	1.09 ± 0.040^a^	1.05 ± 0.002^a^	1.07 ± 0.013^a^	0.96 ± 0.031^ab^	0.99 ± 0.016^a^	1.13 ± 0.028^a^
Chl a/b ratio	Italica	2.19 ± 0.00^ab^	2.22 ± 0.04^ab^	2.18 ± 0.02^ab^	2.31 ± 0.17^a^	2.01 ± 0.17^ab^	2.17 ± 0.07^ab^	2.70 ± 0.30^a^
	Capitata	1.64 ± 0.01^a^	1.69 ± 0.08^a^	1.54 ± 0.01^a^	1.78 ± 0.09^a^	1.59 ± 0.05^a^	1.52 ± 0.05^a^	1.58 ± 0.04^a^
	Botrytis	1.71 ± 0.001^a^	1.70 ± 0.080^a^	1.80 ± 0.009^a^	1.80 ± 0.021^a^	1.94 ± 0.192^a^	1.76 ± 0.070^a^	1.75 ± 0.050^a^
Total carotenoids (µg g^−1^FW)	Italica	5.41 ± 0.05^c^	5.33 ± 0.07^cd^	5.71 ± 0.05^b^	5.89 ± 0.03^a^	5.58 ± 0.06^bc^	5.16 ± 0.03^cde^	5.29 ± 0.07^cd^
	Capitata	10.27 ± 0.26^a^	10.78 ± 0.52^a^	11.05 ± 0.19^a^	10.66 ± 0.82^a^	10.36 ± 0.22^a^	10.69 ± 0.49^a^	10.90 ± 0.45^a^
	Botrytis	8.82 ± 0.14^b^	8.32 ± 0.39^bc^	8.68 ± 0.03^bc^	8.60 ± 0.08^bc^	8.72 ± 0.33^bc^	8.62 ± 0.25^bc^	9.51 ± 0.09^a^

Carotenoid content varied depending on the treatment and concentrations of JA and MeJA used. JA at 1 nM and 1 pM increased the total carotenoid content in *B. oleracea* var. *italica* by 5.49% and 8.82%, respectively, whereas 1 µM MeJA treatment resulted in 3.09% increase compared to that in the untreated control seedlings (Table 2). All other treatments with JA and MeJA reduced total carotenoid content when compared to the controls. In *B. oleracea* var. *capitata*, seed priming with both JA and Me JA led to an increase in total carotenoids, with the highest levels recorded for 1 nM JA (7.62%) and 1 pM MeJA (6.11%), respectively, and when compared to the control (Table 2). The JA and MeJA treatments in *B. oleracea* var. *botrytis* showed effects contrary to that noted in *B. oleracea* var. *italica* and *capitata*. All the three JA treatments (1 µM, 1 nM and 1 pM) led to a reduction in carotenoid content by 5.67%, 1.58%, and 2.52%, respectively, over that in the non-primed control seedlings. In addition, 1 µM and 1 nM Me-JA treatments led to a decrease in carotenoid content by 1.21% and 2.33%, but the seedlings treated with 1 pM had increased carotenoid content by 7.79%, when compared to that in the control seedlings.

### Effect of JA and MeJA on chlorophyll fluorescence

**Figure 1:**
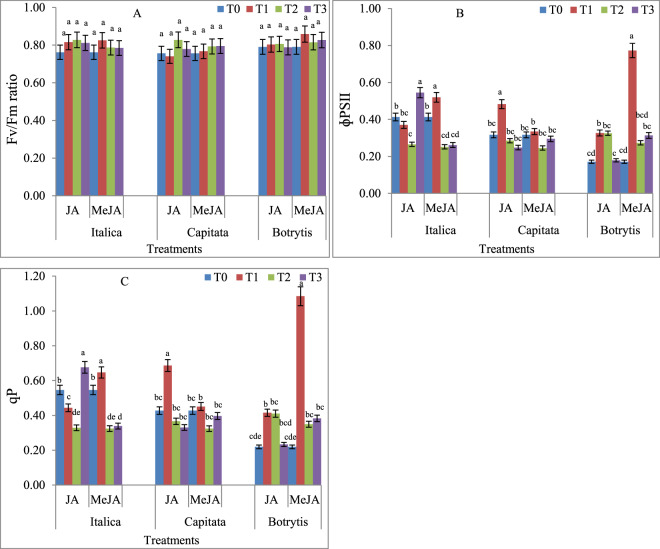
Effects of Jasmonic acid (JA) and Methyl jasmonate (MeJA) on **(A)** Fv/Fm **(B)** ɸPSII and **(C)** qP of *Brassica oleracea* L. Var*. Italica, Capitata, Botrytis*. Values are means ± SD of three independent replications (n = 3). T0-Control, T1- 1 μM JA and Me-JA, T2- 1 nM JA and Me-JA, T3- 1pM JA and Me-JA. Different letters (a-e) within the column indicate statistically significant differences among the treatments, according to Tukey’s test at (P < 0.05).

Various JA and MeJA treatments had non-significant effects on the photosynthetic efficiency of PSII, appraise as *F*v/*F*m ratio. *Brassica oleracea* var. *italica* had the maximum increase in photosynthetic efficiency of PS II when treated with 1 nM JA (8.61%). With 1 µM MeJA treatment, *F*v/*F*m value increased by 8.31%, when compared to the control (Fig. 1A). In *B. oleracea* var. *capitata*, not much effect on photosynthetic efficiency of PSII was observed, the treatment with 1 nM JA and 1 pM MeJA increased the *F*v/*F*m ratio by 9.51% and 5.15%, respectively, when compared to the control (Fig. 1A). On the other hand, in *B. oleracea* var. *botrytis*,1 µM MeJA treatment led to 8.55% increase in *F*v/*F*m value. The JA priming treatment in *B. oleracea* var. *botrytis* did not alter the *F*v/*F*m ratio (Fig. 1A). The photosynthetic yield (ɸ PS II) in JA and MeJA primed treatments in all three varieties varied significantly. *Brassica oleracea* var. *italica* treated with JA had significantly reduced ɸ PS II, with the maximum reduction seen when treated with 1 nM JA (35.73%) compared with the control. However, the treatment with 1 pM JA improved ɸ PS II by 31.85% when compared to the control. The MeJA treatment at 1 µM also improved ɸ PS II by 25.72%, while 1 nM and 1 pM concentrations caused reduction in ɸ PS II by 39.04% and 36.70%, respectively, when compared to the control (Fig. 1B). In *B. oleracea* var. *capitata*, 1 µM JA priming treatment led to a 52.36% increase in ɸ PS II, while 1 µM MeJA treatment resulted only in a minor 5.57% increase over that in the control seedlings. All other JA and MeJA treatments reduced ɸ PS II in *B. oleracea* var. *capitata* compared to the control (Fig. 1B). The MeJA priming treatment was found to be generally effective in increasing ɸ PS II compared to JA in the *B. oleracea* var. *botrytis* seedlings. A notable improvement of 352.04% in ɸ PS II was observed when primed with 1 µM MeJA, compared to that of the control untreated seedlings. (Fig. 1B). Photochemical quenching was improved in the treatments with various doses of JA and MeJA in all three varieties, but the results were inconsistent. These observations show that both derivatives of JAs (JA and MeJA) increased qP in all three varieties. However, MeJA seemed to be more effective in *B. oleracea* var. *botrytis* (Fig. 1C).

### Effect of JA and MeJA priming on Rubisco activity

**Figure 2:**
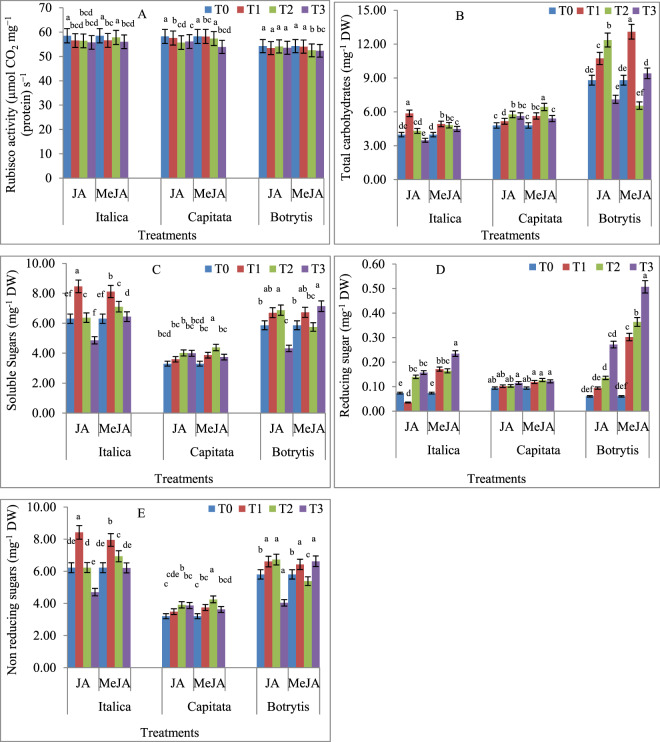
Effects of Jasmonic acid (JA) and Methyl jasmonate (MeJA) on **(A)** Rubisco activity **(B)** Total carbohydrates **(C)** Total soluble sugars **(D)** Reducing sugars and **(E)** Non reducing sugars of *Brassica oleracea* L. Var*. Italica, Capitata, Botrytis*. Values are means ± SD of three independent replications (n = 3). T0-Control, T1- 1 μM JA and Me-JA, T2- 1 nM JA and Me-JA, T3- 1pM JA and Me-JA. Different letters (a-e) within the column indicate statistically significant differences among the treatments, according to Tukey’s test at (P < 0.05).

Rubisco activity did not increase when treated with various doses of JA and MeJA in all three cultivars. In almost all the cases of exogenous JA or MeJA applications, the rubisco activity was found to be decreased. This reduction was, however, statistically non-significant (Fig. 2A).

### Consequence of JA and MeJA priming on total carbohydrates, total soluble sugars, and reducing and non-reducing sugars

Seed priming with JA and MeJA led to a significant accumulation of total carbohydrates in all three cultivars of *B. oleracea* L. The JA and MeJA treatments at 1 µM in *B. oleracea* var. *italic* caused 47.32% and 23.70% increase in total carbohydrate content, respectively, over those in the control seedlings (Fig. 2B). In *B. oleracea* var. *capitata*, the JA and MeJA treatments led to increase in carbohydrate content by 20.37% and 34.07%, respectively, with the highest effect being at 1 nM, compared with that in the control seedlings (Fig. 2B). In *B. oleracea* var. *botrytis*, again 1 nM JA increased the total carbohydrate content by 40.45% over the control seedlings. The MeJA treatment with 1 µM led to an increase in total carbohydrates by 48.79%, compared to the control (Fig. 2B).

Total soluble sugars (TSS) increased due to the exogenous application of JA and MeJA in all three varieties. Maximum TSS accumulation was observed in *B. oleracea* var. *italica* treated with 1 µM of JA or MeJA, which was found to be increased by 34.21% or 28.82%, respectively, when compared to the control (Fig. 2C). In *B. oleracea* var. *capitata*, the treatment with 1 nM JA or MeJA resulted in the highest levels of TSS, which were 21.72% and 32.73%, respectively, when compared to the controls (Fig. 2C). In *B. oleracea* var. *botrytis*, the JA and MeJA treatments at 1 pM resulted in the highest amounts of TSS, which were 26.42% and 21.70%, respectively, compared with the control (Fig. 2C).

The content of reducing sugars (RS) increased many-folds with the JA and MeJA treatments in *B. oleracea* var. *italica* and *botrytis*. The JA and MeJA treatments to seeds at 1 pM led to an increase of 113.57% and 218.55% in RS, in the plants raised from the primed seeds, respectively, compared to that in the control seedlings raised from non-primed seeds (Fig. 2D). In *B. oleracea* var. *capitata*, JA and MeJA treatments had no effect on the RS content, unlike observed in *B. oleracea* var. *italica* and *botrytis*, but 1 pM of JA and MeJA increased RS content by 21.98% and 29.43%, respectively, compared to that of the control (Fig. 2D). The RS levels in *B. oleracea* var. *botrytis* varied significantly depending on the concentration of JA and MeJA applied. For instance, 1 pM of JA and MeJA increased RS by 349.72% and 740.88%, respectively, compared to the control (Fig. 2D).

Non-reducing sugar (NRS) content increased in *B. oleracea* var. *italica*, when treated with JA and MeJA. In the 1 µM treatment, the NRS content in the seedlings increased by 35.35% and 27.74%, respectively, compared with the control (Fig. 2E). In *B. oleracea* var. *capitata*, exogenous application of both JA and MeJA increased the NRS content., and the highest increase was observed when treated with 1 nM JA and 1 nM MeJA, with values of 22.16% and 32.67%, respectively, compared with the control (Fig. 2E). In *B. oleracea* var. *botrytis*, JA and Me-JA treatments led to an increase in NRS, albeit to a lesser extent. The maximum increase of 15.97% over control was observed in the seedlings treated with 1 nM JA. For MeJA, treatment with 1 pM resulted in the highest increase (14.13%) of NRS, and when compared to the control (Fig. 2E).

### Effects of JA and MeJA priming on the expression of *PsbI, PsbL, PsbM, PsbTc(p)* and *PsbTc(n)* genes

**Figure 3:**
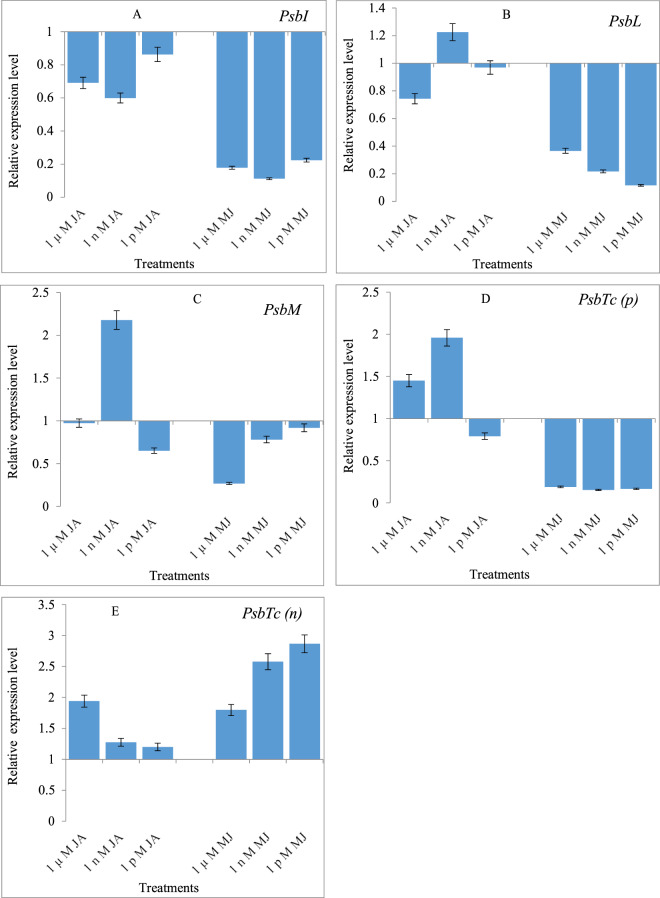
Effects of Jasmonic acid (JA) and Methyl jasmonate (MeJA) on Gene expression of **(A)** *PsbI* **(B)** *PsbL* **(C)** *PsbM* **(D)** *PsbTc(p)* and **(E)** *PsbTc (n)*of *Brassica oleracea* L. Var*. Italica*.

**Figure 4:**
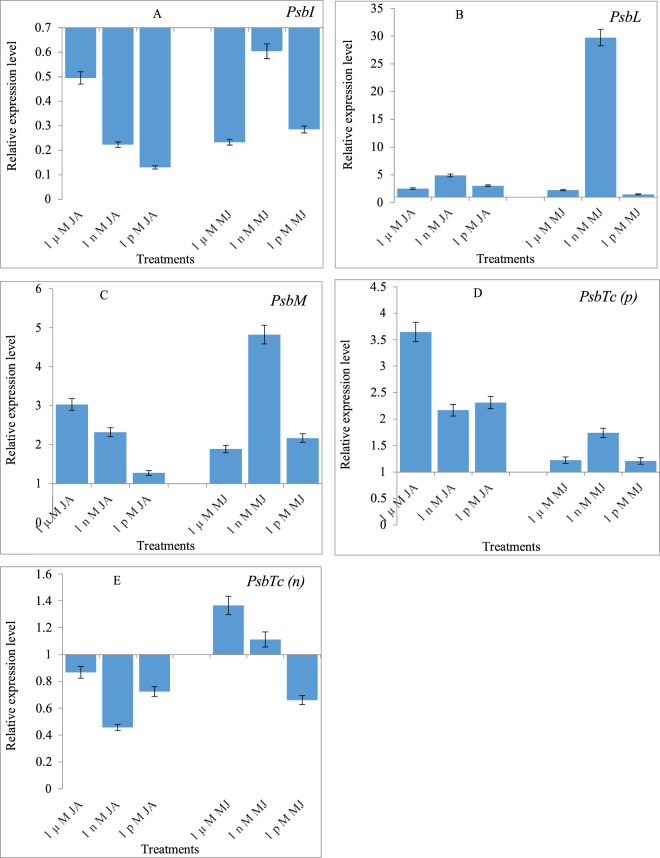
Effects of Jasmonic acid (JA) and Methyl jasmonate (MeJA) on Gene expression of **(A)** *PsbI* **(B)** *PsbL* **(C)** *PsbM* **(D)** *PsbTc(p)* and **(E)** *PsbTc(n)*of *Brassica oleracea* L. Var*. capitata*.

**Figure 5:**
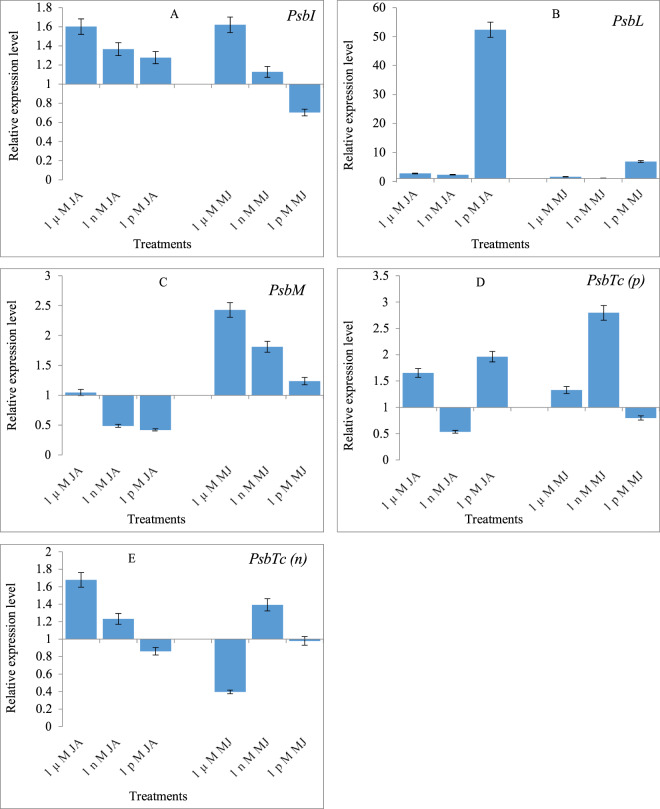
Effects of Jasmonic acid (JA) and Methyl jasmonate (MeJA) on Gene expression of **(A)** *PsbI* **(B)** *PsbL* **(C)** *PsbM* **(D)** *PsbTc(p)* and **(E)** *PsbTc(n)* of *Brassica oleracea* L. Var*. botrytis*.

A few genes of PSII were examined for their expression in 10-day old seedlings of *B. oleracea* L. var. *italica*, *capitata* and *botrytis*, raised from seeds primed with JA and MeJA. In *B. oleracea* L. var. *italica* the expression of *PsbI* gene was down-regulated when treated with JA or MeJA, compared to the *PsbI* expression in the control seedlings (Fig. 3A). In *B. oleracea* L. var. *capitata*, JA and Me-JA downregulated the *PsbI* expression (Fig. 4A). However, in *B. oleracea* L. var. *botrytis*, the *PsbI* expression was found to be upregulated in all three different treatments, which was different from that observed in *B. oleracea* L. var. *italica* and *capitata*. Maximum upregulation of gene expression occurred with 1 µM, followed by 1 nM, and 1 pM JA. MeJA also resulted in the upregulation of gene expression in the 1 µM and 1 nM treatments, with the maximum values obtained with 1 µM treatment. At the lowest concentration of Me-JA (1 pM), the *PsbI* expression was significantly down-regulated (Fig. 5A).

In *B. oleracea* L. var. *italica*, the expression of *PsbL* was upregulated 1.225-fold with 1 nM JA, compared to that in the control seedlings, but it was downregulated in the highest (1 µM) and lowest (1 pM) concentration of JA. At the same time, the downregulation of gene expression was noted in all MeJA priming treatments (Fig. 3B). In *B. oleracea* L. var. *capitata*, the treatment with JA and MeJA at all three concentrations upregulated the expression of *PsbL* gene. Priming with 1 nM JA and MeJA resulted in a maximum upregulation of gene expression by 4.902 and 29.719 folds over that in the control seedlings (Fig. 4B). In *B. oleracea* L. var. *botrytis*, JA and MeJA led to the upregulation of the expression of *PsbL* in all three priming treatments. JA and MeJA at the lowest concentration (1 pM) had the maximum upregulation of the *PsbL* gene expression by 52.346 and 6.869 folds compared to the control (Fig. 5B).

JA treatment (1 nM) in *B. oleracea* L. var. *italica* led to upregulation in the expression of *PsbM* gene by 2.178-fold compared to the control seedlings. The treatment with MeJA led to downregulation of the *PsbM* gene expression in all priming treatments, and this increase had been in a dose-dependent manner (Fig. 3C). In *B. oleracea* L. var. *capitata*, both JA and MeJA treatments led to the upregulation of the *PsbM* gene expression. Maximum upregulation was observed in the seedlings resulting from the 1 µM-primed seeds, and it was upregulated by 3.031-fold compared to the control. Similar upregulation up to 4 or 5-fold was observed for the *PsbM* gene expression in the seedlings raised from the seeds primed with1 nM MeJA (Fig. 4C). In *B. oleracea* L. var. *botrytis*, MeJA showed a stronger effect than did JA in upregulation of the *PsbM* gene, and was upregulated by 1.045-fold in the seedlings raised from 1 µM MeJA-primed seeds compared with the control. The MeJA treatment at 1 pM had the highest upregulation of the *PsbM* gene expression, which increased by 2.428-fold compared to the control (Fig. 5C).

In *B. oleracea* L. var. *italica*, the *PsbTc(p)* gene was upregulated by the JA treatments, which increased in a dose-dependent manner from 1 µM (1.451 fold) to 1 nM (1.959 fold), over the control. However, the lowest concentration of JA (1 pM) led to downregulation of the *PsbTc(p)* gene expression. Concurrently, MeJA has an inhibitory effect, as all the three concentrations resulted in downregulating the expression of *PsbTc(p)* (Fig. 3D). In *B. oleracea* L. var. *capitata*, both JA and MeJA treatments led to the upregulation of *PsbTc(p)* gene expression; however, the 1 µM JA treatment had a stronger effect, and increased the expression by 3.647-fold over the control. In comparison, 1 nM of the MeJA treatment resulted only in moderate upregulation of the *PsbTc(p)* gene by 1.741-fold (Fig. 4D). In *B. oleracea* L. var. *botrytis*, JA priming upregulated the *PsbTc(p)* gene expression by 1.963-fold, and the maximum effect was observed when treated with 1 pM, compared with the control. The MeJA treatment with 1 nM resulted in a maximum upregulation of the *PsbTc(p)* gene expression by 2.796-fold over the control (Fig. 5D).

The *PsbTc(n)* gene expression in *B. oleracea* L. var. *italica*, was found to be upregulated with the JA and MeJA treatments. However, the MeJA treatments showed the opposite trend of upregulation. The JA treatment (1 µM) resulted in the upregulation of the *PsbTc(n)* gene expression by 1.941-fold, compared to the control, but the treatment with MeJA (1 pM) resulted in a maximum of this gene by 2.868-fold, compared with the control (Fig. 3E). In *B. oleracea* L. var. *capitata*, the JA treatment downregulated the expression of *PsbTc(n)* at all three concentrations tested. On the other hand, MeJA upregulated the *PsbTc(n)* gene expression by 1.366 and 1.112 folds, at 1 µM and 1 nM treatments, respectively, compared to the control (Fig. 4E). In *B. oleracea* L. var. *botrytis*, the *PsbTc(n)* gene expression was upregulated following the treatment with 1 µM and 1 nM, by 1.678 and 1.231-fold when compared to the control. The treatment with 1 nM of MeJA resulted in the upregulation of the *PsbTc(n)* gene expression by 1.392-fold, compared with the control (Fig. 5E).

## Discussion

Exogenous application of JA and MeJA had a strong influence on root length in all three cultivars of *B. oleracea* L.; particularly, in *B. oleracea* var. *capitata*, the root length increased by 58.68% compared to the control. Suppression of the inhibitory effect of exogenous application of JA and MeJA on primary root growth has been studied by overexpressing of NINJA and JAZ proteins responsible for the deletion, mutation, or variation in the JA domain^25^. While this might be applicable in our study, however, further validation is necessary. Unlike the root, shoot length decreased with JA and MeJA treatments, despite JA reportedly mimics auxin-like activity in regulating plant growth. The inhibitory effect of the two JA conjugates was dose-dependent, and it is being first time observed that the JAs counteraction also depends on the genetic makeup of plants, as the three cultivars of *B. oleracea* L. performed differently for shoot inhibition, and maximum inhibition was observed in *B. oleracea* var*. italica*. This behaviour of JA is because of the fact that it regulates shoot length by inhibiting mitotic cell division; cell elongation and cell division^26^. MeJA application to *A. thaliana* suspension culture repressed the activation of M phase genes and arrested the cell in G_2_ phase^26,27^. Both the moisture content and the fresh weight increased due to the application of JA or MeJA. Dry weight increased mostly due to the application of JAs. Mir *et al*.^28^ recorded similar results in maize plants, in which seeds affected by salinity were pre-treated with JA, after which plant growth and biomass were found to be increased.

Exogenous application of JAs enhanced photosynthetic pigments and improved the photosynthetic efficiency. However, these changes were in a dose-dependent manner. Total chlorophyll content increased the most in *B oleracea* L. *italica*, by 7.8%, particularly when treated with 1 pM JA. In *B. oleracea* L*. capitata*, it increased by 5.73% when treated with 1 nM JA. Enhanced content of chlorophyll pigments might have been due to improvement in the activities of the enzymes like proto-chlorophyllide reductase and α-aminolevulinic acid dehydratase involved in the biosynthesis of chlorophyll^29–31^. There are two major enzymes that regulate chlorophyll biosynthesis in higher plants, i.e. α-aminolevulinic acid dehydratase and proto-chlorophyllide reductase^31^. α-aminolevulinic acid dehydratase is a common precursor of the tetrapyrrole ring in chlorophyll structure and is known to alleviate certain stress conditions by enhancing chlorophyll synthesis^29^. On the other hand, the proto-chlorophyllide reductase is also well known to enhance the chlorophyll content of higher plants by the light-dependent protochlorophyllide reduction^30,32^. Our results contradict the findings of Cotado *et al*.^20^, which reported a reduction in total chlorophyll content when treated with jasmonates in *Saxifraga longifolia*^20^. While the effects of JA could be correlated to the accumulation of carotenoids in *B oleracea* L. *italica* and *capitate*. However, the carotenoid levels in *B oleracea* L. *botrytis* remained unchanged. Sirhindi *et al*. also observed an increase in carotenoid content due to the exogenous application of JA^33^. The relationship between photosynthesis and JA is known^32^, and the interaction of transcription factor JAZs with YABBY1 and YABBY3, which are involved in JA signalling leading to increased chlorophyll degradation^34^. JA signalling may work independent of the COI1-JAZs-MYC2/3/4 signalling cascade in *B. oleracea* L., however, this needs further confirmation^35^. The Chl a/b ratio increased due to exogenous priming with JA and MeJA in *B. oleracea* L., var. *italica* and *botrytis*, and to some extent in *capitata*. These findings which support the view that JA enhances the photosynthetic efficiency by increasing chlorophyll a content. Chl a is the immediate donor of photon to the reaction centres of PS II and PSI. Our results are in agreement with those of Cotado *et al*.^20^. Increase in chl a over chl b and to some extent the carotenoids, facilitate in maintaining the photosynthetic complex at a higher level, which further leads to an increase in sugars, particularly after treating with JA and MeJA. These results align with those of Attaran *et al*.^36^, who described a close relationship between the PS II quantum efficiency and expression levels of the genes involved in growth and photosynthesis^36^. No changes were observed in RuBisCO from the exogenous application of JA and MeJA in all *B. oleracea* cultivars. Some authors have reported a decline in RuBisCO activity due to JA or MeJA, quite similar to our findings^37^. PÉRez and Goossens^38^ described the physiological role of JA-Ile by activating the JA signalling pathway in *Arabidopsis*. Based on varying results obtained, it is possible that different JA signalling pathways exist for JAs, and may be independent of the COI1- MYC2- JAZ co-receptor pathway^39^. Application of JA and MeJA resulted in *F*v/*F*m ratio of around 0.83, indicating no photoinhibition in *B. oleracea* L. as compared to the control. In *B. oleracea* L. var. *italica*, the photosynthetic efficiency of PS II increased due to JA and MeJA, but no changes were observed in *capitata*. Our results are similar to those reported in some other studies that JA promotes *F*v/*F*m, thereby indicating a maximal quantum efficiency of PS II^33,40,41^. Further studies are needed to investigate this in detail.

A significant increase in total carbohydrates was observed in all three cultivars of *B. oleracea* L. due to the application of JA or MeJA, which showed a considerable involvement of these conjugates in CO_2_ fixation by these plants. Similarly, total soluble sugars also increased after treatment with JA and MeJA in all three cultivars of *B. oleracea* L. Accumulation of reducing sugars and to some extent non-reducing sugars by JA and MeJA reflects a regulatory role of the conjugates in photosynthetic metabolism as indicated from the observations recorded in our study. The activation level of RuBisCO in the present study after the application of JA and MeJA also supports the higher accumulation of photosynthetic products in all three cultivars of *B. oleracea* L., as RuBisCO is responsible for limiting photosynthetic CO_2_ assimilation, and its reduced level would be responsible for wiping off the limitation in photosynthetic CO_2_ assimilation. Our results are in agreement with those of Guo, *et al*.^42^ who have reported increased RuBisCO activity that limited the photosynthetic CO_2_ assimilation and photorespiration in MeJA-treated Jin1-9/myc2 plants.

A few genes involved in the regulation of PS II activity were examined after the exogenous application of JA or MeJA. We observed that *PSbI* was down-regulated in both *italica* and *capitata* by the JA and MeJA treatments, while it was up-regulated in *botrytis*. Both *PsbM* and *PsbI* are required for the efficient formation and stability of PSII dimers *in vivo*. They have different roles; PsbM being located in the center is required directly for the formation of dimers and its absence can lead to the instability of the dimers accumulated^13,43^. In the present study, while comparing the PS II efficiency with the expression of *Psb* (ɸ PSII) genes in *botrytis*, it was confirmed that JA and Me-JA treatment improved both function and stability of PSII, which is required for ɸ PSII enhancement. In *capitata*, *PsbM* showed up-regulation while *PsbI* showed down-regulation. In all the treatments of JA and Me-JA, a consistency with the photosynthetic efficiency as well as qP, i.e. photochemical quenching, being directly dependent on the formation and stability of PS II dimmer, was observed. In *italica* both *PsbI* and *PsbM* were down-regulated except at 1 nM JA where *PsbM* showed more than two-fold up-regulation. Uto *et al*.^43^ have reported the functional role of *PsbM* in the stabilization of the PSII dimer and maintenance of electron transfer efficiency of PSII based on X-ray crystal structure analysis at a resolution of 4.2 Å. It was found that deletion of *PsbM* results in a slight widening of the lipid hole involving QB, which induces structural changes of the bicarbonate ion coordinated to the non-heme Fe(II) atom, and destabilizes the polypeptide chains around the QB binding site located far from the position of *PsbM* and decrease in the electron transfer rate from QA to QB in *PsbM* deletion mutants i.e. ∆PsbM-PSII as compared to the wild-type PSII which was interpreted on basis of structural changes caused by the deletion of the *PsbM* subunit. In the present study, gene expression of *psbI* and *psbM* in different concentrations of JA and Me-JA showed a direct co-regulation with PSII (ɸ PSII) efficiency as well as photochemical quenching in all three cultivars. Analysis of expression of *PsbM* in tobacco plants unveiled its prominent role in altering the properties of the Q_B_ site and the electron flow within PSII rather than in the biogenesis of higher order PSII complexes in plants^12^. These differences in results suggested the presence of different signalling pathways of JAs which either involved *MAPKs* or *Ca*^*2+*^*-calmodulin* or some other protein kinases involvement might be possible in regulating gene expression responsible in PSII RC protein synthesis in different cultivars of *B. oleracea*. Due to this reason, dynamic regulations of the *PSbI* gene have been observed. Another gene *PsbL*, responsible for reduction reactions of PSII and Q_A_ activity as an electron acceptor, was found to be upregulated when treated with 1 nM JA, but down-regulated with 1 µM and 1 pM concentrations of JA. In *italica, PsbL* expression was up-regulated at 1 nM JA concentration, while down-regulation was recorded in 1 µM and 1 pM concentrations of JA. This unveiled accountability of JA for photosynthetic machinery could also be supported by the physiological results explained above. *Capitata* and *botrytis* also showed up-regulation of *PsbL*, thereby strengthening the photosynthetic efficiency as well as CO_2_ fixation in these two cultivars by both JA conjugates. The electron flow in PS II as well as the dimerization of the PSII reaction centre is influenced by *PsbM*, which was found to be up-regulated in all three cultivars. *PsbTc* gene, responsible for repairing PSII from photodamage, facilitating the binding of Q_A_ with it and its reactivation after photoinhibition damages, was up-regulated when supplemented with JA or MeJA in most of the cultivars studied. PsbTc is located close to PsbM and PsbL in the dimerization domain^3^ and the deletion of its gene destabilises the binding of PsbM and results in monomerization of the dimeric PSII core^44^. Ohnishi and Takahashi^45^ revealed the role of this protein in the efficient post-translational repair process of photo-damaged PSII under high light conditions by comparing wild-type and a PsbTc-deficient mutant in green alga *Chlamydomonas reinhardtii*. More recently, its role in the early assembly steps of PSII has been shown^46^. Directed inactivation of PsbTc in tobacco plants did not prevent photoautotrophic growth, but clearly affected the PSII forward and backward electron transfer reactions, stability of PSII dimers and the assembly of PSII light-harvesting complexes (LHCII)^14^.

## Conclusion

Photosynthesis is an important metabolic process, which provides chemical energy by using sunlight for all forms of life on earth. PSII protein complex is the most important component of the photosynthetic apparatus. Its synthesis, biogenesis and management are crucial for photosynthetic efficiency, which are ultimately responsible for photosynthetic productivity in the form of different saccharides. Several reviews are available in the literature regarding the PSII assembly and repair mechanism, but as per our knowledge, no single research paper is available which discusses the assembly and stability of genes responsible for efficient formation and stability of PSII. Furthermore, no reports are available to date which would discuss the effect of plant growth regulators in maintaining the structural stability and functional efficiency of PSII in any particular crop plant. The present results suggest that Me-JA at low concentrations has more potential in making the PSII structure more stable and actively efficient as compared to JA. Besides, the genetic makeup of different varieties also matters in showing the effect of exogenous application of JA and Me-JA, which is very clear in the present study wherein three cultivars showed variable performance for the *Psb* gene expression as well as for photosynthetic efficiency. However, further biochemical and molecular studies are required to elucidate the adaptation of dynamic nature of PS II towards changing environmental conditions, particularly in the context of achieving enhanced crop productivity.

## Material and methods

### Experimental set up

Viable seeds of broccoli (*Brassica oleracea*L. var. *italica*, *capitate* and *botrytis*) were sterilized by immersing in 0.01% HgCl_2_ solution for 2 min. After washing with tap water 3–4 times, the seeds were pre-soaked for 6 hrs in solution containing different concentrations (1 μM, 1 nM and 1 pM) of JA and MeJA. The seeds were arranged *viz* Control; 1 μM MeJA; 1 μM JA; 1 nM MeJA; 1 nM JA; 1pM Me-JA and 1pM JA. After pre-soaking the seeds were allowed to germinate in Petri plates containing Whatman’s filter paper, for 3 days in a growth chamber (indosaw) with 25 °C, photoperiod of 16 h dark/light period and 80% humidity. Low light (LL) stress were initiated by providing light with the intensity of 200 μmol (photon m^−2^s^−1^). The experiment was laid in randomised block design with three replicates each. The seedlings were shifted to the growth paper for 10 days after appearance of plumule. After that the seedlings were harvested for the analysis of total chlorophyll, chla/b, carotenoids, maximum quantum yield of PSII photochemistry (Fv/Fm), effective quantum yield of PSII photochemistry (ΦPSII), photochemical (qP,) non-photochemical quenching (NPQ), Rubisco activity, total carbohydrates, total soluble sugars, reducing and non-reducing sugars, and gene expression by using RT PCR.

### Growth and biomass yield

Root and shoot length were measured manually using a scale. Dry weight was recorded after drying the sprouts at 70 °C for 48 h in an oven.

### Chlorophyll and carotenoid content

Chlorophyll and carotenoids were extracted from known weight of fresh first (primary) leaf using 80% acetone. The extract was used to determine absorbance at 645, 663 and 470 nm using a spectrophotometer (Beckman 640 D, USA)^40^.

### Chlorophyll fluorescence

Chlorophyll fluorescence parameters of the first (primary) leaf was determined using a portable pulse amplitude-modulated fluorimeter (Heinz Walz). The method of White and Critchley^47^ was used. A dark period of 20 min was provided to the plants before measuring. Using a modulated radiation not sufficient to induce any photosynthesis in the leaf, the minimum fluorescence (F_0_) was calculated for all PSII reaction centres (RCs) that were open. The maximal fluorescence (F_m_) was measured when all RCs were closed, using a 0.8 s pulse of saturating radiation of 3000 µmol m^−2^s^−1^ in leaves acclimatized to dark.

### Rubisco activity (EC 4.1.1.39)

Using the method of Usuda^48^, ribulose 1,5-bisphosphatecarboxylase/oxygenase activity was determined by monitoring NADH oxidation at 30 °C at 340 nm. Leaf tissue (1 g) was homogenized using a chilled mortar and pestle with ice-cold extraction buffer containing 0.25 M Tris-HCl (pH7.8), 0.05 M MgCl_2_, 0.0025 M EDTA, and 37.5 mg DTT. The homogenate was centrifuged at10,000×g for 10 min at 4 °C. The resulting supernatant was used for the enzyme assay. The reaction mixture (3 mL) contained 100 mM Tris-HCl (pH 8.0), 40 mM NaHCO_3_, 10 mM MgCl_2_, 0.2 mM NADH, 4 mM ATP, 5 mM DTT, 1U of glyceraldehyde 3-phosphodehydrogenase, 1 U of 3-phosphoglycerate kinase, and 0.2 mM ribulose 1,5-bisphosphate (RuBP).

### Total carbohydrates, soluble sugars, reducing and non-reducing sugars

The protocol of DuBois, *et al*.^49^ was used to estimate total carbohydrates. The OD was taken at 485 nm using a spectrophotometer (Beckman 640 D, USA). Total soluble sugars were estimated as described by Loewus^44^. The absorbance was recorded at 630 nm. The estimation of reducing sugars was done by adopting the procedure of Nelson-Somogyi^50^ using a spectrophotometer (Beckman 640 D, USA) and determining absorbance at 620 nm. Non-reducing sugar content was calculated by deducting the amount of reducing sugars from the total soluble sugars and expressed as mg/g DW tissue.

### Gene expression analysis

All the three varieties supplemented with JA and MeJA were further analysed by real time polymerase chain reaction (RT-PCR, Applied Biosystems). Total RNA was extracted from the leaves using GET^TM^ Total RNA Kit. RNA concentration and purity were determined at 260 and 280 nm using the nanodrop. The first strand cDNA was prepared from 1 µg RNA template using miRNA 1^st^-Strand DNA Synthesis Kit. cDNA was amplified using specific primers (Table 3). The relative abundance of β-actin (AB047313) was also determined in order to standardise the results, which was defined as 100 relative expression units (REU) and used as the internal standard. The relative expression ratio of each gene was calculated using the comparative Ct value method^51^. The experiments were repeated twice independently, and the fold change was calculated. The relative expression value with fold change of ≥2.0 and ≤0.5 as compared to control untreated seedlings gene expression was considered to be upregulated and downregulated, respectively. Gene expression was analysed using REST.

**Table 3:** Sequence of forward and reverse primers used in gene expression analysis.

Sr. No.	Gene Name	Primer Name	Sequence (5′-3′)
1.	*PsbI*	*BopsbiFw:*	CAAACTTTTTGTATACACTGTAG
		*BopsbiRe:*	TTATTCTTCACGTCCCGGATT
2.	*PsbL*	*BopsblPf:*	TGACACAATCAAATCCGAACG
		*BopsblRe:*	TCGAAAATAAAACAGCAAGTACA
3.	*PsbM*	*BopsbmPf:*	AATATTCTTGCATTTATTGCTACT
		*BopsbmRe:*	TTAATCATTTTGACTAACGGTTTT
4.	*PsbTc(p)*	*Bopsbtc(p)Pf:*	TGGAAGCATTGGTTTATACATTT
		*Bopsbtc(p)Re:*	TTTTAGGTGGTTCCCGAAAAAA
5.	*PsbTc(n)*	*Bopsbtc(n)Pf:*	GGCTGAAGAAGAAGAGCCC
		*Bopsbtc(n)Re:*	CAGTAGCGGCAGATCTTGG

### Statistical analysis

The data presented here was subjected to one-way analysis of variance (ANOVA) to analyse the effects of different concentrations of JA and MeJA. The results are expressed as the mean ± standard error of three replicates. Tukey’s test (P < 0.05) was applied for the multiple comparison using Graph Pad Prism Version 7.
